# Patterns and predictors of anxiety medication use among United States adults: a national analysis with a focus on racial disparities in the Asian population

**DOI:** 10.3389/fpubh.2026.1803386

**Published:** 2026-05-15

**Authors:** Jingjing Gao, Muinat Abolore Idris, Sung In H. Kim-Vences, Jason H. Windett

**Affiliations:** 1 Department of Management, Policy, and Community Health, University of Texas Health Science Center at Houston School of Public Health, Houston, TX, United States; 2 Department of Health Promotion and Behavioral Sciences, School of Public Health, University of Texas Health Science Center at Houston, Houston, TX, United States; 3 Department of Family and Community Medicine, Baylor College of Medicine, Houston, TX, United States; 4 Department of Medicine, Baylor College of Medicine, Houston, TX, United States; 5 School of Data Science, Department of Political Science and Public Administration, Public Policy Ph.D. Program, University of North Carolina at Charlotte, Charlotte, NC, United States

**Keywords:** anxiety disorders (health sciences), anxiety medication, Asian Americans, mental health, minority health and mental health

## Abstract

**Background:**

Anxiety disorders are highly prevalent in the United States, but treatment utilization varies substantially across racial and ethnic groups. Asian adults are known to underutilize mental health services despite experiencing psychological distress, but evidence on medication-specific patterns remains limited. The goal of this study is to quantify differences in anxiety medication use, especially among Asian Americans, and to evaluate the factors associated with their lower use of medication for anxiety disorders.

**Methods:**

We analyzed data from the 2023 National Health Interview Survey (NHIS; *N* = 29,466) using survey-weighted logistic regression models to estimate population-level associations. Adults who reported having ever been diagnosed with an anxiety disorder (*n* = 5,431) made up the initial eligible group. After excluding respondents without complete data, 3,118 adults remained in the full analytic sample used for survey-weighted logistic regression models 1 and 2. A separate sub-sample of Non-Hispanic Asian adults with complete data (*n* = 87) was analyzed independently.

**Results:**

Medication use varied significantly by race. Non-Hispanic White adults reported the highest use (64.3%), while Asian adults reported the lowest (45.1%), despite having a history of anxiety disorder. In fully adjusted models, Asian adults had significantly lower odds of medication use compared with White adults (OR = 0.55, 95% CI [0.32, 0.93], *p* < 0.05). Race × age interactions were not statistically significant, but predicted probability plots showed a declining trend of medication use with age among Asian adults, contrasting with increasing use among White adults. Subsample analyses of Asian adults showed few significant predictors due to the small sample size, although higher income was linked to increased medication use.

**Conclusion:**

Asian adults in the United States have significantly lower odds of anxiety medication use. Findings highlight ongoing disparities that may stem from cultural stigma, structural barriers, and unequal access to mental health care. Targeted efforts to provide culturally responsive anxiety treatments for Asian communities are necessary.

## Introduction

Anxiety disorders are among the most common mental health issues in the United States, impacting roughly one in five adults each year (1, 2). Effective treatments such as psychotherapy and medication (mainly anxiolytics and antidepressants) are available, but substantial disparities remain in access to mental health care. Population-level data consistently reveal wide differences in medication use among various demographic, socioeconomic, geographic, and cultural groups. Racial and ethnic minority groups often face reduced access and lower utilization of treatment even when their symptom severity is similar to or greater than that of non-Hispanic White adults (3–5). Understanding the predictors of anxiety medication use is therefore essential for identifying inequities in mental health care access and guiding interventions aimed at increasing treatment uptake among social minority populations, especially by developing policies targeted at vulnerable groups.

Asian Americans are among the fastest-growing racial groups in the United States, yet they consistently have the lowest rates of mental health service utilization. Previous research has connected these lower odds of medication use to cultural stigma around mental illness (6–8), language barriers (6, 9–11), which can be due to a shortage of culturally matching providers, and possible differences in how symptoms are expressed. However, there is limited national data specifically on the use of anxiety medications among Asian adults. Most studies do not separate medication use from broader mental health service patterns, which limits our understanding of anxiety treatment disparities within this population.

Age, gender, and structural factors are known to influence mental health treatment behaviors, including the use of psychotropic medication. While anxiety disorders can affect individuals at any age, younger adults tend to use medications less often than middle-aged and older adults. This pattern may be due to higher stigma, less interaction with health providers, and a stronger preference for non-drug therapies (12–16). Older adults are more likely to receive psychotropic medications due to higher healthcare utilization and a greater burden of chronic symptoms, although age patterns may differ across cultural and racial groups (12, 13). Among Asian American adults, qualitative and community-based studies reveal strong stigma surrounding psychiatric medication in older generations, leading to avoidance of pharmacologic treatment (17, 18). Gender differences are also well documented: women have significantly higher rates of psychotropic medication use than men, mainly because of higher diagnosis rates and greater help-seeking behavior (19–21). Having insurance can reduce financial barriers, but it does not automatically translate to higher mental health service use among Asian Americans (7, 10, 11). Even with coverage, Asian Americans have some of the lowest rates of mental health service use compared to other groups, with only 8.6% seeking services versus nearly 18% of the general United States population (7, 22).

Despite socioeconomic advantages in income and education, Asian Americans show lower treatment rates, termed the “paradox of high education but low utilization” (23). Factors like BMI are included as covariates because they are associated with health care utilization patterns, especially as individuals with higher BMI often have more frequent clinical encounters, which increase the chance for mental health screening and medication prescription (24). Geographic regions with strong mental health infrastructure still face treatment gaps (25, 26). Social factors like marital status influence help-seeking; some Asian cultures see mental illness as a private family matter, discouraging external help (27). Education negatively predicts mental health medication use (28), and Asian Americans, a highly educated racial group, deserve further study as a vulnerable subgroup in mental health medication utilization. Few national studies examine factors that predict anxiety medication use among Asian Americans, leaving their demographic and socioeconomic influences under-investigated.

Using nationally representative data from the 2023 National Health Interview Survey (NHIS), this study investigates racial differences in anxiety medication use among United States. adults with a history of anxiety. We aim to (1) describe the demographic and socioeconomic characteristics of adults with anxiety, including an Asian subgroup; (2) identify predictors of anxiety medication use across the entire sample; and (3) examine whether age-related patterns in medication use differ by race, with particular focus on Non-Hispanic Asian adults. Through both population-wide and subgroup analyses, this study aims to generate new insights into structural and cultural factors that may contribute to disparities in anxiety medication use among Asian Americans.

## Methods

### Data source

We performed a cross-sectional analysis using data from the 2023 NHIS, which is a nationally representative sample of the United States civilian, noninstitutionalized population. The NHIS, conducted annually by the National Center for Health Statistics, employs a multistage, stratified cluster sampling design to collect data on health conditions, healthcare use, and demographic and socioeconomic factors.

### Study population

**Figure 1:**
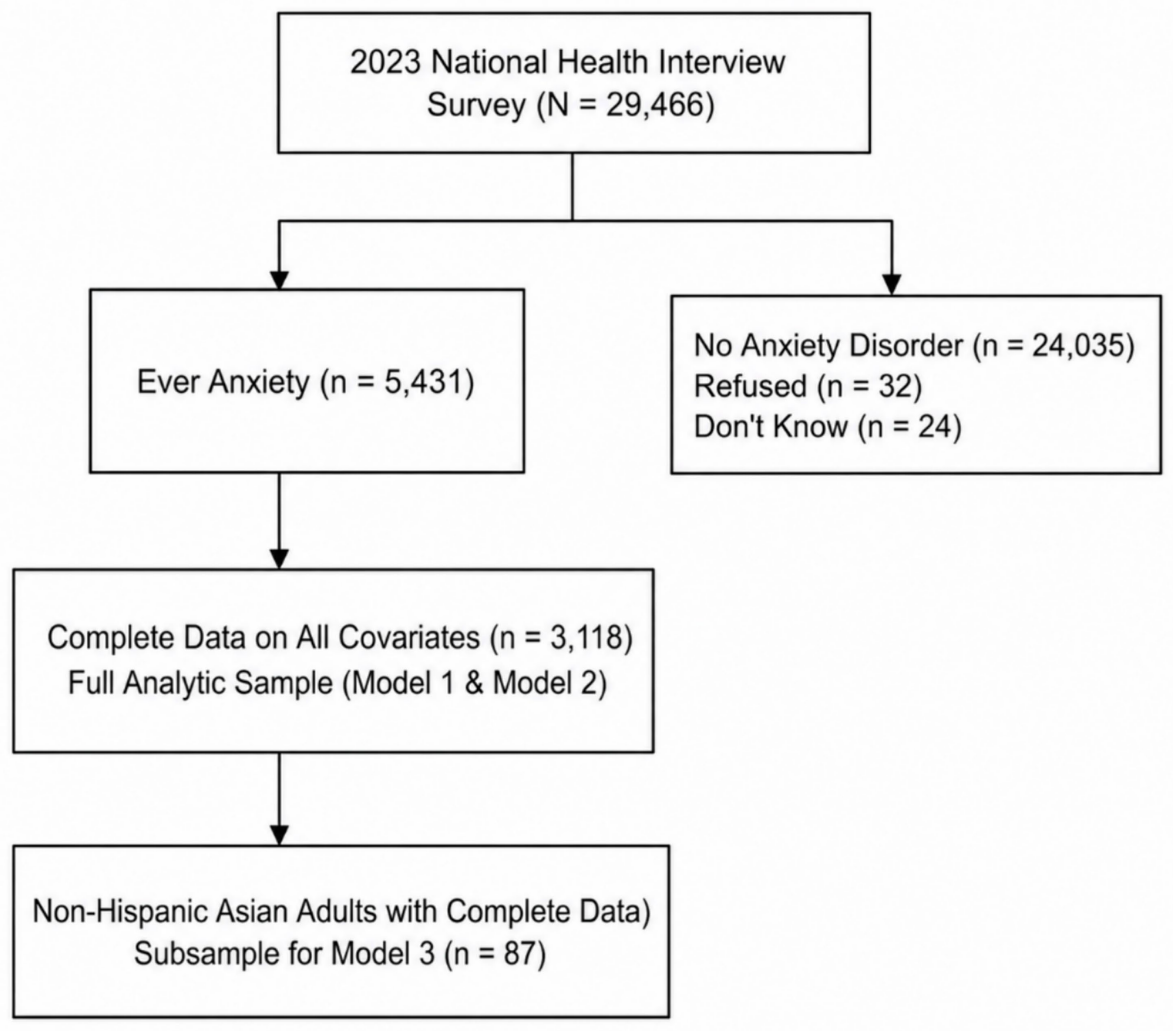
Flow diagram of study participants for anxiety medication use analyses in the 2023 national health interview survey, including Asian subsample identification.

We used data from the 2023 NHIS (*N* = 29,466), and of all adult respondents, 5,431 reported ever having been told by a health professional that they had an anxiety disorder. Individuals without an anxiety diagnosis (*n* = 24,035), which includes those who responded “refused” (*n* = 32) and “do not know” (*n* = 24), were excluded from the analytic cohort. Among adults with a history of anxiety, 3,118 had complete data on all demographic, socioeconomic, and health-related covariates and were included in the full analytic sample for Models 1 and 2, whereas 2,313 were excluded due to missing values for the core covariates. To examine racial subgroup patterns, we also identified a subsample of Non-Hispanic Asian adults with complete covariate information (*n* = 87), which was analyzed in Model 3. The resulting samples allowed for both population-level estimates and a focused assessment of anxiety medication use within the Asian population. Figure 1 shows the detailed flow diagram of study participants for anxiety medication use.

### Measures

The primary outcome was current anxiety medication use, defined from the NHIS item asking whether respondents with diagnosed anxiety were “now taking prescription medication for anxiety.” Responses were coded dichotomously (1 = yes, 0 = no). The main predictors were race/ethnicity, which were categorized as: “Non-Hispanic White,” “Hispanic,” “Non-Hispanic Black,” “Non-Hispanic Asian,” and “Non-Hispanic American Indian/Alaska Native (AIAN) or Other.” Covariates were selected based on prior literature demonstrating their relevance to mental health treatment utilization. These included: (1) Sex (male, female); (2) Health insurance coverage (insured vs. uninsured); (3) Poverty ratio, a continuous measure of income relative to the federal poverty level; (4) Age, as continuous variable; (5) United States census region (Northeast, Midwest, South, West); (6) Marital status (married vs. not married); (7) Education (<high school, high school/GED, some college, bachelor’s degree, graduate/professional degree; and (8) Body Mass Index (BMI) category (normal weight, overweight, obesity class I, obesity class II/III). This was included as a control variable because individuals with higher BMI generally have more frequent clinical encounters, which may increase opportunities for mental health screening and treatment. To examine whether age differentially affected medication use across racial groups, we estimated a race × age interaction.

### Statistical analysis

All statistical analyses were conducted using Stata version 18 (StataCorp LLC) (29). Survey design elements (weights, strata, and primary sampling units) were applied using svy commands to produce population-level estimates. Logistic regression was used because the outcome (medication utilization) is binary complete covariate data (30–32).

### Descriptive analysis

We first generated weighted descriptive statistics for the full analytic sample and the Asian-only subsample. For categorical variables, weighted frequencies and percentages were reported; for continuous variables, weighted means and standard deviations were provided.

### Multivariable regression

We estimated three survey-weighted logistic regression models: (1) Model 1 (Full Sample): Adjusted for all demographic, socioeconomic, and health-related covariates; (2) Model 2 (Full Sample with Interaction): Added a race × age interaction term to evaluate whether age-related differences in medication use varied by race; and (3) Model 3 (Asian Subsample): Replicated Model 1 covariates within the Non-Hispanic Asian subsample to examine within-group predictors. Regression coefficients were reported as log-odds, with statistical significance defined as *p* < 0.05.

### Predicted probability plots

To visualize differences in medication use by race across age we generated predicted probabilities of anxiety medication use from Model 2 for ages 20 to 80. Although interaction terms were not statistically significant, plots provided meaningful descriptive insight into racial patterns. Confidence intervals were derived using survey-adjusted post-estimation procedures.

### Ethics approval and consent to participate

This study was based on publicly available, de-identified NHIS data. In accordance with federal regulations and University policy, this research was considered exempt from human subjects review.

## Results

Table 1 shows the characteristics of the full sample of adults with complete covariate data on anxiety history (*N* = 3,118) and the Non-Hispanic Asian subgroup used in Model 3 (*N* = 87). In the full sample, the average age was 41.2 years, while it was 38.5 years in the Asian subgroup. Women made up about two-thirds of the full sample (67.2%) and a similar percentage of the Asian subgroup (70.1%). Insurance coverage was generally high, especially among Asian adults (79.3% compared to 69.8% in the full sample). The Asian subgroup also had a higher average poverty ratio (5.27 versus 4.33), indicating relatively higher household income compared to federal poverty levels. Notable differences appeared in body weight and geographic distribution. Over half of non-Hispanic Asian adults were classified as overweight (51.7%), a significantly higher rate than in the full sample (30.3%), while fewer Asian adults fell into Obesity I or II + categories. Regionally, nearly half of the Asian subgroup lived in the West (44.8%), compared to 24.6% in the overall sample. Education levels also varied; Asian adults had higher representation in bachelor’s (35.6%) and graduate degree categories (19.5%) compared to the full sample (26.8 and 13.9%, respectively). Overall, these descriptive findings show that the Asian subgroup tends to be younger, more insured, more highly educated, and more regionally concentrated, emphasizing the importance of subgroup-specific analyses as in Model 3.

**Table 1:** Descriptive characteristics of the full analytic sample and the non-hispanic Asian subsample, national health interview survey (NHIS) 2023.

Variable	Full sample (*n* = 3,118)					Asian subsample (*n* = 87)				
	Count (percentage)	Mean	SD	Min	Max	Count (percentage)	Mean	SD	Min	Max
Age		41.16	14.21	18	83		38.52	13.69	19	80
Sex (1 = male, 2 = female)										
Male	1,022 (32.8%)					26 (29.9%)				
Female	2096 (67.2%)					61 (70.1%)				
Race (indicator variables)										
NH White	2,345 (75.2%)									
Hispanic	362 (11.6%)	0.11	0.32	0	1					
NH Black	218 (7.0%)	0.07	0.25	0	1					
NH Asian	87 (2.8%)	0.02	0.16	0	1		1	0	1	1
NH AIAN	106 (3.4%)	0.03	0.18	0	1					
Has insurance (1 = yes)	2,177 (69.8%)	0.69	0.45	0	1	69 (79.3%)	0.79	0.40	0	1
Poverty ratio		4.32	2.91	0	11		5.27	3.07	0	11
BMI (indicator variables)										
Overweight	944 (30.3%)	0.30	0.46	0	1	45 (51.7%)	0.51	0.50	0	1
Obesity I	920 (29.5%)	0.29	0.45	0	1	25 (28.7%)	0.28	0.45	0	1
Obesity II+	1,199 (38.4%)	0.38	0.48	0	1	15 (17.2%)	0.17	0.38	0	1
Region (indicator variables)										
Midwest	794 (25.5%)	0.25	0.43	0	1	9 (10.3%)	0.10	0.30	0	1
South	1,071 (34.3%)	0.34	0.47	0	1	25 (28.7%)	0.28	0.45	0	1
West	768 (24.6%)	0.24	0.43	0	1	39 (44.8%)	0.44	0.5	0	1
Married (1 = yes)	1,209 (38.8%)	0.38	0.48	0	1	41 (47.1%)	0.47	0.50	0	1
Education (indicator variables)										
High school/GED	571 (18.3%)	0.18	0.38	0	1	14 (16.1%)	0.16	0.37	0	1
Some college	964 (30.9%)	0.30	0.46	0	1	15 (17.2%)	0.17	0.38	0	1
Bachelor’s	837 (26.8%)	0.26	0.44	0	1	31 (35.6%)	0.35	0.48	0	1
Graduate	432 (13.9%)	0.13	0.34	0	1	17 (19.5%)	0.19	0.39	0	1
Professional/doctoral	130 (4.2%)	0.04	0.2	0	1	9 (10.3%)	0.10	0.30	0	1

Values are survey-weighted using national health interview survey (NHIS) design variables (weights, strata, and primary sampling units).

**Figure 2:**
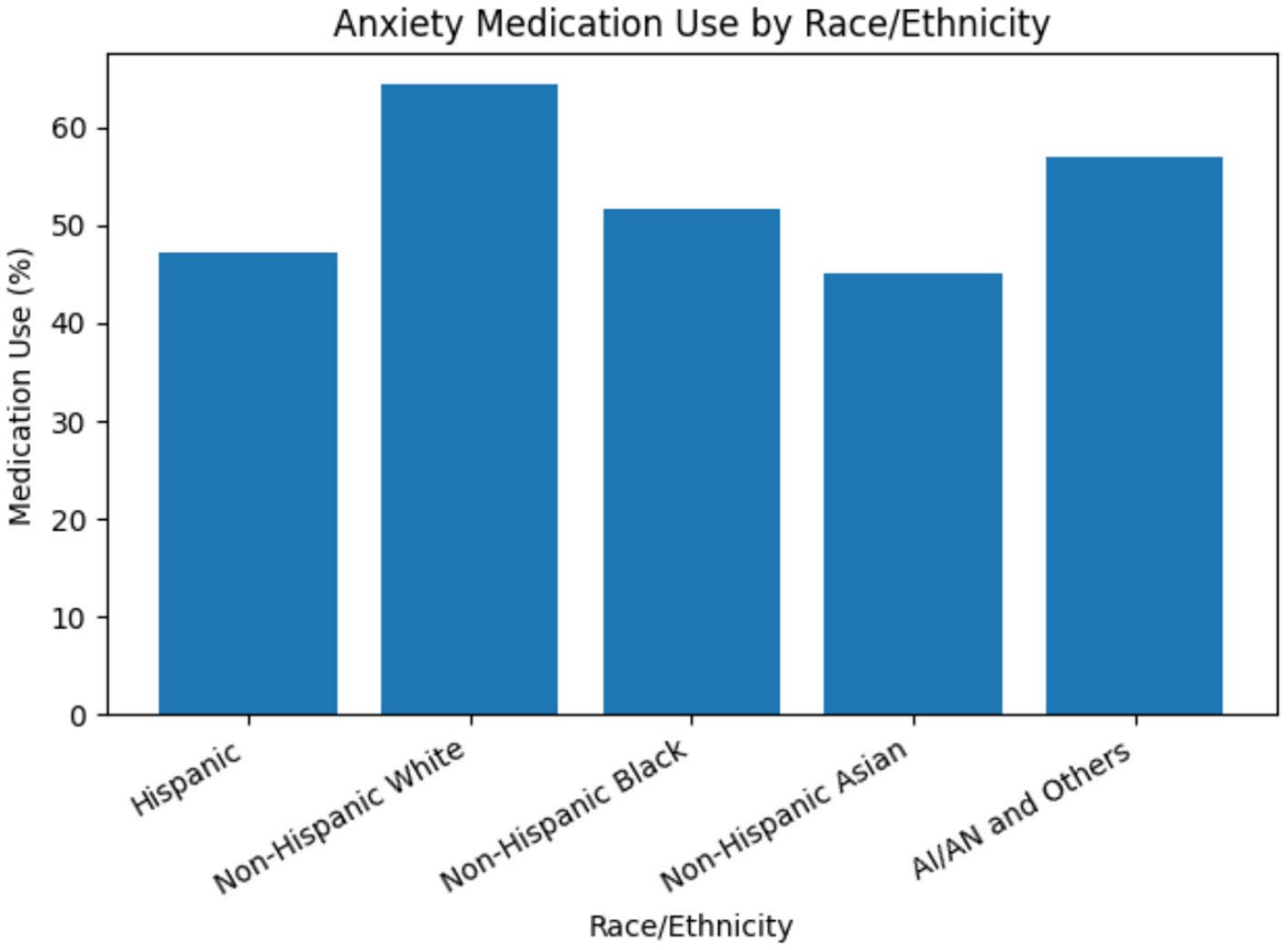
Weighted percentage of United States adults with a history of anxiety reporting current use of prescription medication for anxiety, by race/ethnicity, NHIS 2023.

Among United States. adults with a history of anxiety, medication use varied significantly by race (design-based *F* (3.95, 2407.27) = 16.16, *p* < 0.05). Non-Hispanic White adults had the highest prevalence of anxiety medication use (64.3%), followed by American Indian/Alaska Native adults (57.0%) and Black adults (51.6%), based on Table 2. Hispanic adults (47.1%) and Asian adults (45.1%) were least likely to use anxiety medication despite reporting anxiety, as shown in Figure 2. These findings suggest meaningful racial disparities in access to or utilization of mental health treatment among individuals with anxiety.

**Table 2:** Lower anxiety medication use among asian adults relative to other racial groups.

Race	Medication (%)	No medication (%)
Hispanic	47.13	52.87
Non-hispanic white	64.31	35.69
Non-hispanic black	51.64	48.36
Non-hispanic asian	**45.06**	**54.94**
AI/AN and others	56.98	43.02
Total	60.41	39.59

Table 3 presents the survey-weighted logistic regression results examining factors associated with anxiety medication use among United States adults with a history of anxiety. In the full sample (Model 1), age was positively associated with medication use (OR = 1.01, 95% CI [1.01, 1.02], *p* < 0.001), and women had significantly higher odds of using anxiety medication than men (OR = 1.52, 95% CI [1.28, 1.81], *p* < 0.001). Several racial and ethnic minority groups showed lower odds of medication use relative to non-Hispanic White adults. Notably, non-Hispanic Asian adults had significantly lower odds of medication use (OR = 0.55, 95% CI [0.32, 0.93], *p* < 0.05), indicating a disparity in treatment use among individuals with a history of anxiety.

**Table 3:** Survey-weighted logistic regression predicting anxiety medication use among United States adults with ever anxiety.

Variable	Model 1: full sample (*n* = 3,118)	Model 2: race × age (*n* = 3,118)	Model 3: Ssian subsample (*n* = 87)
Age	1.01 [1.01, 1.02]***	1.02 [1.01, 1.02]***	0.98 [0.94, 1.02]
Female (ref = male)	1.52 [1.28, 1.81]***	1.52 [1.28, 1.81]***	0.91 [0.22, 3.70]
Race (ref = NH White)			
Hispanic	0.50 [0.37, 0.65]***	0.49 [0.21, 1.12]†	
NH Black	0.54 [0.38, 0.77]**	0.56 [0.20, 1.55]	
NH Asian	0.55 [0.32, 0.93]*	1.29 [0.25, 6.55]	
NH AIAN	0.69 [0.41, 1.17]	1.50 [0.30, 7.44]	
Race × Age			
Hispanic × Age		1.00 [0.98, 1.02]	
NH Black × Age		1.00 [0.98, 1.02]	
NH Asian × Age		0.98 [0.94, 1.02]	
NH AIAN × Age		0.98 [0.94, 1.02]	
Has insurance	0.93 [0.77, 1.13]	0.93 [0.77, 1.13]	1.55 [0.40, 5.95]
Poverty ratio	1.03 [0.99, 1.06]	1.03 [0.99, 1.06]	1.35 [1.10, 1.66]**
BMI (ref = normal)			
Overweight	0.82 [0.42, 1.58]	0.81 [0.42, 1.56]	0.55 [0.04, 8.31]
Obesity I	1.12 [0.57, 2.19]	1.10 [0.56, 2.15]	0.82 [0.05, 13.51]
Obesity II+	1.15 [0.58, 2.28]	1.13 [0.57, 2.24]	0.63 [0.03, 14.10]
Region (ref = Northeast)			
Midwest	1.12 [0.87, 1.45]	1.13 [0.88, 1.45]	0.06 [0.00, 1.22]†
South	1.08 [0.83, 1.39]	1.08 [0.84, 1.39]	1.16 [0.18, 7.60]
West	0.89 [0.67, 1.18]	0.89 [0.67, 1.18]	0.51 [0.07, 3.79]
Married	0.96 [0.80, 1.15]	0.96 [0.80, 1.15]	0.66 [0.16, 2.72]
Education (ref = <HS)			
High school/GED	1.76 [1.18, 2.64]**	1.75 [1.17, 2.62]**	3.32 [0.32, 34.80]
Some college	1.81 [1.24, 2.63]**	1.80 [1.24, 2.61]**	2.71 [0.32, 23.00]
Bachelor’s	2.24 [1.51, 3.31]***	2.23 [1.51, 3.29]***	2.25 [0.31, 16.43]
Graduate	2.17 [1.41, 3.33]***	2.15 [1.40, 3.31]***	1.32 [0.16, 10.71]
Professional/doctoral	1.78 [1.04, 3.06]*	1.78 [1.04, 3.07]*	

Values are odds ratios (OR) with 95% confidence intervals. Models are survey-weighted using NHIS design variables. Model 2 includes interaction terms between race and age. Significance levels: *p < 0.05, **p < 0.01, ***p < 0.001, †*p* < 0.10.

**Figure 3:**
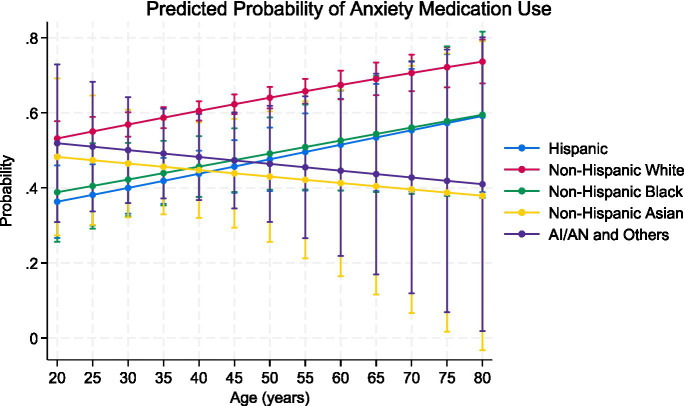
Predicted likelihood of anxiety medication use by race/ethnicity across age groups in United States adults with a history of anxiety. Predicted probabilities were derived from the survey-weighted logistic regression model (Model 2) that included race, age, and their interaction, while adjusting for sex, insurance status, poverty ratio, BMI category, region, marital status, and education. Vertical bars represent 95% confidence intervals.

Model 2 incorporated interactions between race and age to assess whether age-related patterns in medication use varied across racial groups. None of the interaction terms were statistically significant, and these results should therefore be interpreted as exploratory. However, the predicted probabilities shown in Figure 3 illustrate descriptive differences across racial groups. Among non-Hispanic White adults, the probability of anxiety medication uses increases with age, rising from approximately 0.55 at age 20 to nearly 0.75 by age 80. Hispanic and non-Hispanic Black adults show a more gradual increase across the lifespan, with both groups converging around a probability of 0.55–0.60 in later adulthood. In contrast, non-Hispanic Asian adults exhibit a declining probability of medication use with age, decreasing from approximately 0.47 at age 20 to below 0.40 by age 80. Although these patterns are not statistically significant, they suggest potential differences in age-related treatment use that warrant further investigation. Estimates for AI/AN and other racial groups should be interpreted with caution due to the small sample size (6, 9–11) and wide confidence intervals.

Overall, Model 2 and the accompanying figure indicate that racial disparities in anxiety medication use persist across the observed age range, and Asian adults show the most consistent lower odds of medication use patterns, with probabilities that remain lower than those of other groups across all ages and decline further in older adulthood. These results suggest potential cultural, structural, or access-related factors influencing treatment use among Asian adults that may intensify with age. Although these patterns are not statistically significant (all *p* > 0.05), they should be interpreted as descriptive and hypothesis-generating rather than confirmatory (Figure 3).

Model 3 focused specifically on the Asian subsample (*n* = 87). Estimates were imprecise due to the small sample size and should be interpreted with caution. Age was not significantly associated with medication use (OR = 0.98, 95% CI [0.94, 1.02], *p* = 0.38), and neither sex (OR = 0.91, 95% CI [0.22, 3.70], *p* = 0.896) nor insurance coverage (OR = 1.55, 95% CI [0.40, 5.95], *p* = 0.53) were significant predictors. The only statistically significant factor was poverty ratio (OR = 1.35, 95% CI [1.10, 1.66], *p* < 0.01), indicating that higher-income Asian adults were more likely to report current use of anxiety medication. Regional differences were observed but were not statistically significant; for example, Asian adults in the Midwest had lower estimated odds of medication use compared to those in the Northeast (OR = 0.06, 95% CI [0.00, 1.22], *p* < 0.10), although this estimate was imprecise and should be interpreted cautiously. Given the small sample size, results from the Asian subsample should be considered exploratory and interpreted with caution.

Taken together, the results indicate a persistent racial disparity in the treatment of anxiety, with Asian adults significantly less likely to use anxiety medications, even after accounting for socioeconomic, demographic, and regional factors. This pattern remained evident in subgroup analysis, highlighting potential cultural, structural, and access-related barriers that warrant further investigation.

## Discussion

These findings reflect differences in current anxiety medication use among individuals with a prior diagnosis of anxiety and should not be interpreted as direct evidence of unmet clinical need or longitudinal patterns of treatment use. This study demonstrates an apparent and persistent racial disparity in anxiety medication use in the United States (7, 23, 33–35). Non-Hispanic White adults reported the highest levels of anxiety medication use (64.31%), whereas Non-Hispanic Asian adults reported the lowest (45.06%), despite all individuals having been diagnosed with anxiety at some point in their lives. Furthermore, Asian adults had the lowest rates of medication use, even after controlling for factors like age, sex, socioeconomic status, insurance, region, and education. These disparities persisted through predicted probability estimates, which showed decreasing medication use with age among Asian adults, unlike the increasing trend seen in non-Hispanic White adults.

Various factors may contribute to these patterns. Cultural stigma around psychiatric medication is a recognized obstacle in many Asian communities and can become more pronounced with age. For example, older Asian adults might prefer somatic explanations for psychological issues or opt for non-drug approaches for emotional health. Structural barriers also influence these trends. Although many Asian adults in our study have insurance and higher education levels, these benefits do not always lead to fair access to culturally sensitive mental health services. A shortage of providers who speak the same language, a lack of awareness of treatment options, and beliefs that mental health care is culturally inappropriate can all further decrease medication use. These findings may also be understood through the lens of social cognitive and cultural theories of health behavior (36, 37). Concepts such as self-efficacy and learned helplessness suggest that individuals’ beliefs about their ability to manage symptoms or benefit from treatment can influence medication use (38). In addition, theories of independent and interdependent self-construal highlight how cultural norms shape help-seeking behavior, particularly in collectivist contexts where mental health concerns may be managed within the family rather than through formal medical treatment (39, 40). These theoretical perspectives provide a framework for understanding how cultural beliefs and behavioral expectations may contribute to observed differences in treatment use.

The subgroup analysis, although constrained by a small sample size, offers important insights. Among Asian participants, a higher poverty ratio-reflecting greater household income-was linked to a greater likelihood of medication use, implying that economic resources can influence treatment engagement (41–45). Regional differences also emerged as significant: Asian adults in the Midwest had notably lower predicted medication use compared to those in the Northeast, though the estimates were imprecise. These findings suggest that local health system features, community stigma, and geographic access to culturally appropriate providers may all play a role in shaping treatment decisions (23, 46).

### Limitations

There were a few limitations: (1) the models used anxiety medication as a proxy for what is more crucial-symptom management or control. Long-term use of medications like benzodiazepines can be harmful, and prioritizing non-pharmacologic interventions might be beneficial. (2) There was selection bias, partly because the study only included participants with a prior diagnosis of anxiety disorder, excluding those with undiagnosed anxiety. (3) The sample had low Asian representation, possibly due to the second issue, even though Asians were a key population of interest.

## Conclusion

These findings highlight the importance of implementing culturally tailored public health strategies. Such strategies could include community-based education to combat stigma, greater integration of behavioral health services into primary care settings that serve Asian populations, and expanding access to mental health providers who match patients’ cultural and linguistic backgrounds. Future research should also explore the diversity within Asian subgroups, an area not fully addressed by NHIS, to uncover specific cultural or structural reasons behind the lower odds of medication use and mental health services. Overall, this study reveals a significant treatment gap for Asian Americans suffering from anxiety disorders. Closing this gap will require collaborative efforts from clinical, community, and public health sectors to ensure Asian adults receive timely, appropriate, and culturally sensitive anxiety treatment.

## Data Availability

The original contributions presented in the study are included in the article/supplementary material, further inquiries can be directed to the corresponding author.
